# Enterovirus D68 – The New Polio?

**DOI:** 10.3389/fmicb.2018.02677

**Published:** 2018-11-13

**Authors:** Hayley Cassidy, Randy Poelman, Marjolein Knoester, Coretta C. Van Leer-Buter, Hubert G. M. Niesters

**Affiliations:** Department of Medical Microbiology and Infection Prevention, Division of Clinical Virology, University of Groningen, University Medical Center Groningen, Groningen, Netherlands

**Keywords:** Enterovirus D68, emerging, pathogenicity, surveillance, outbreak response

## Abstract

Enterovirus D68 (EV-D68) has emerged over the recent years, with large outbreaks worldwide. Increased occurrence has coincided with improved clinical awareness and surveillance of non-polio enteroviruses. Studies showing its neurotropic nature and the change in pathogenicity have established EV-D68 as a probable cause of Acute Flaccid Myelitis (AFM). The EV-D68 storyline shows many similarities with poliovirus a century ago, stimulating discussion whether EV-D68 could be ascertaining itself as the “new polio.” Increasing awareness amongst clinicians, incorporating proper diagnostics and integrating EV-D68 into accessible surveillance systems in a way that promotes data sharing, will be essential to reveal the burden of disease. This will be a necessary step in preventing EV-D68 from becoming a threat to public health.

## Introduction

### Enterovirus D68: The Virus

Enterovirus D68 is a single-stranded positive-sense RNA virus of the *Picornaviridae* family, belonging to the species enterovirus D. EV-D68 was first isolated from respiratory samples in 1962 in California, United States from four pediatric patients presenting with acute respiratory symptoms. The four isolates obtained from the patients were referred to as the Fermon, Franklin, Robinson,and Rhyne strains, each presenting with similar antigenic properties. As a representative strain of the new serotype, the Fermon strain was selected (Imamura and Oshitani, 2015). Since its first description, EV-D68 has been classified into three genetic clades, A, B and C. Subclades A1, A2, B1, and B2 have evolved and can be further identified depending on enterovirus typing, targeting either VP1 or VP4-2 capsid protein (Nix et al., 2006; Esposito et al., 2015). EV-D68 was also previously known as rhinovirus 87 until it was re-classified in 2002 (Blomqvist et al., 2002). EV-D68 is unusual in that it has shared characteristics from two key members of the *Picornaviridae* family; enterovirus and rhinovirus. Firstly, it has a lower optimal growth temperature of 33°C (the temperature of the nose), allowing better replication in the nasal cavity than other EV, and secondly it is acid sensitive, meaning it is unable to adequately survive during passage in the stomach (Foster et al., 2015). Phylogeny however, shows that EV-D68 is genetically more closely related to EV than to rhinoviruses. Studies into the cellular receptors of EV-D68 have shown that it targets the α2-6-linked sialic acid, which is present on cells in the upper respiratory tract, indicating tropism toward this area (Imamura et al., 2014). This critical difference in tropism is significant when comparing EV-D68 to poliovirus, which predominantly reproduces in the gastrointestinal tract. It is likely that most EV-D68 infections are asymptomatic or present with a mild respiratory illness however, it is difficult to know it’s true circulation and burden on the community. Nevertheless, studies have indicated that circulation of EV-D68 increases over the summer-autumn season like with other EV (Tokarz et al., 2012).

### The Beginning of Enterovirus Awareness – The Polio Era

Enteroviruses are thought to have existed and coevolved with humanity for thousands of years. One of the oldest records of enterovirus is an Egyptian carving thought to illustrate a priest with a small, weakened limb, which is considered a typical feature of a past polio infection. The causative agent of poliomyelitis (poliovirus), was not discovered until 1908 (Paul, 1971). It was not only the first enterovirus to be discovered, but also caused the most devastating and widespread morbidity and mortality of all the enterovirus genotypes. Poliovirus infection can result in a variety of symptoms, of which AFP that can cause lifelong disability and may result in death, is the most typical clinical entity. Awareness of polio increased during the 20th century. This is largely attributed to President Franklin Roosevelt, himself paralyzed from polio, who was instrumental in founding the National Foundation for Infantile Paralysis which started mass worldwide vaccination campaigns (Noor and Krilov, 2016). The first poliovirus vaccine was an inactivated injectable vaccine, developed by Jonas Salk in 1955. The second vaccine administered orally, was a vaccine developed by Albert Sabin in 1961 (Noor and Krilov, 2016). These primary awareness programs, together with the vaccination campaign, initiated in the fifties, paved the way for the GPEI created in 1988, which aimed to eradicate the virus. Over the subsequent years, following the initial discovery of poliovirus, over 100 enterovirus serotypes have now been discovered with nearly 70 species infecting humans (Craighead, 2000). Non-polio EV can cause a variety of clinical syndromes, ranging from hand-foot and mouth disease to aseptic meningitis.

sThe introduction of the poliovirus vaccine dramatically reduced the incidence of infections globally, with only small clusters sporadically occurring. According to the GPEI, only two countries, Afghanistan and Pakistan still report endemic wild-type poliovirus in circulation in 2018 (The Global Poliovirus Eradication Initiative [GPEI], 2018). Recently, the GPEI also reported a few new cases of vaccine-derived poliovirus in the Democratic Republic of the Congo, Nigeria, Somalia and Papua New Guinea (The Global Poliovirus Eradication Initiative [GPEI], 2018). Therefore, cases of AFP outside these countries have decreased to very low numbers. Remaining cases of AFP are also linked to GBS and neurological infections caused by other viruses such as WNV and more recently, non-polio EV.

### The Rise in Awareness of EV-D68

**FIGURE 1:**
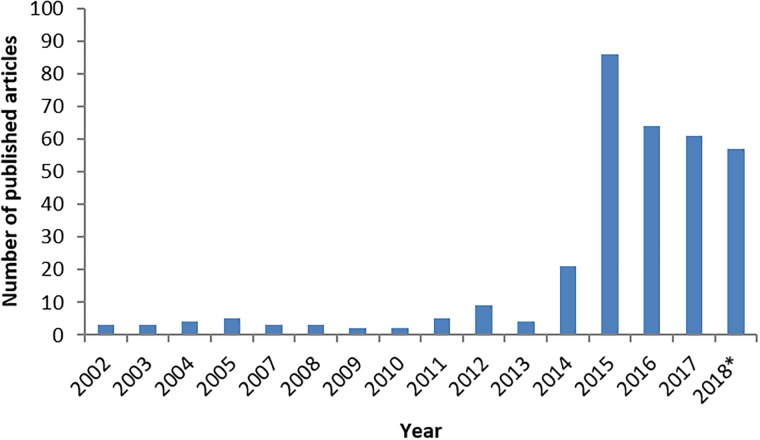
The number of published articles on PubMed describing Enterovirus-D68 from January 2002 to October2018. The number of published articles for each year is in accordance with EV-D68 interest from the previous year. ^∗^Until October.

In the majority of patients, EV-D68 only causes mild respiratory illness. However, the co-occurrence of EV-D68 and a predominantly severe respiratory disease on one side and neurological complications of “polio-like” paralysis on the other side has established EV-D68 as an emerging pathogen (Holm-Hansen et al., 2016). While EV-D68 has been known as a respiratory pathogen since its first description in 1962, the apparent change in pathogenicity into a virus capable of causing AFP over a relatively short period of time, has led to increased interest and awareness of the virus in recent years. This is reflected in the number of published papers. Figure 1 reveals the result of a PubMed search for Enterovirus-D68. With the disappearance of poliovirus as a major threat to public health, enterovirus networks such as the ENPEN, have been set up along with established networks such as the ESCV to focus on non-polio EV which may become new challenges (Harvala et al., 2018). EV-D68 has become a compelling topic for research over the recent years, due to a mix of increased prevalence, pathology and awareness. This review will explore the EV-D68 story further.

### Epidemiology: From Sporadic Respiratory Virus to Emerging Neuropathogenic Threat

Enterovirus D68 had only been reported sporadically worldwide since 2010. Indeed before 2014, only sporadic outbreaks were reported in the US, the Philippines, Japan and the Netherlands (Rahamat-Langendoen et al., 2011; Gong et al., 2016). Significantly, there were only 26 cases reported in the United States between 1970 and 2005 and only 699 cases described in Europe, Southeast Asia and Africa between 1970 and 2013 (Holm-Hansen et al., 2016; Wang et al., 2017). However, it must be noted that specific testing for EV-D68 or routine typing for EV was and is not standard practice in the majority of laboratories and assuredly not for testing respiratory samples. Therefore the true burden of disease is not known and trends have been likely to be missed. A study published in 2012 reports the analysis of trends in EV-D68 circulation across the United States and in Africa over two decades (Tokarz et al., 2012). This was a direct result of increasing sporadic reports of respiratory disease associated with EV-D68 across North America, Europe and Asia. The study found the genome had undergone a rearrangement from the initial Fermon strain in the spacer region of the 5′UTR, which is known to affect the translational efficiency and thought to increase the virulence. Further rearrangement led to a separation into clades A, B and C, with additional deletions within each clade. Interestingly, another study (Kaida et al., 2017) reported clade B had specific substitutions in the BC-loop, which is found on the surface canyon of the VP1 protein, known to have a role in antigenicity. This evolution may have had significant implications in the run up to the 2014 outbreak, but remains unclear so far.

#### The 2014 Outbreak

In August 2014, the United States and Canada experienced the first few cases of what resulted in the largest known EV-D68 outbreak in history. An unprecedented number of cases, particularly in young children, of severe-respiratory illness was linked to EV-D68 infections. Unexpectedly, this outbreak of severe respiratory infections coincided with an upsurge in AFP. Many of the affected children were also shown to have concurrent EV-D68 infections. The Polio and Picornavirus Laboratory Branch of the CDC tested 56 respiratory samples and found EV-D68 to be the most commonly detected pathogen, with an overall rate of 20% (11/56) in samples tested. Furthermore the detection increased to 47% (8/17) in samples collected near onset of respiratory illness (≤7 days) (Sejvar et al., 2016). Fifty-five CSF specimens were also tested and found to have one positive EV-D68 sample (also positive for Epstein-Barr virus) (Sejvar et al., 2016). The paralysis seen in patients infected with EV-D68 was clinically defined as AFM which was essentially, an acute onset of AFP with MRI scans showing motor neuron damage in the myelum. This will be discussed more in detail later in see section “EV-D68 Case Definition.” In 2014, in the United States alone, 120 children were reported with AFM which met the case definition (Messacar et al., 2016). Furthermore, over a 1000 hospital admissions and 12 deaths were associated with an EV-D68 respiratory infection (Levy et al., 2015). A subsequent study found that most EV-D68 positive samples associated with AFM, clustered into the B1 subclade. Interestingly, the study found that five out of six coding polymorphisms present in the subclade B1 strains were associated with neuropathogenic poliovirus (Greninger et al., 2015). These results suggested that the virus had changed in pathogenicity since the originally isolated Fermon strain, a hypothesis which was also supported by results of studies in mice models (Hixon et al., 2017b), which will be discussed later.

At that time, severe respiratory outbreaks of EV-68 were reported from several countries across Europe in 2014. During these outbreaks, cases of AFM were identified, including one in France and in the United Kingdom and two in Norway (Lang et al., 2014; Pfeiffer et al., 2015; Varghese et al., 2015; Holm-Hansen et al., 2016). It is still unclear how frequently EV-D68 causes AFM compared to the number of respiratory infections caused by this virus. Estimating this frequency is currently impossible, firstly as the background circulation of this relatively emerging virus is unknown and secondly, only children with severe respiratory illness were tested in the 2014 outbreak in the United States and in Canada, therefore there is no information on the frequency at which EV-D68 causes mild symptoms. One study investigated EV-D68 detection by country, mostly in Northern and Western Europe, during the United States outbreak (Poelman et al., 2015b). Out of 17,248 respiratory (majority) specimens tested, 4273 had confirmed picornavirus detection with 389 samples positive for EV-D68. In Southern and Eastern Europe, too few samples were tested to draw any conclusions. Hence, crucial information about how large the threat of EV-D68 could be, is still missing.

Regarding poliomyelitis, studies have shown that 1 in 200 poliovirus infections led to irreversible paralysis, with 5–10% of paralysis cases resulting in death due to breathing difficulties (World Health Organization [WHO], 2018). As AFP is not a reportable disease in many countries, as long as it is not caused by poliovirus, it is impossible to say how many potential cases of EV-D68 associated AFM have occurred during upsurges of EV-D68 in the past few years. As the link between EV-D68 and AFM had not been established at that time, many children who presented with sudden paralysis were not adequately sampled to detect EV-D68. Subsequent data from the EV-D68 outbreak in the United States and Canada in 2014 indicates that neurological complications could occur in 1 out of 100 symptomatic cases with a total of 1153 confirmed EV-D68 respiratory infections, and 12 EV-D68 positive AFM cases during this period (Sejvar et al., 2016). Similarly, in Europe, out of 389 confirmed positive samples in 2014, four AFM cases and one death were associated with EV-D68 (Poelman et al., 2015b; Varghese et al., 2015). Limited EV-D68 detection was seen both in the United States and in Europe in 2015 (Wang et al., 2017).

#### The 2016 Outbreak

In 2016, a new upsurge in the number of EV-D68 cases was first reported from the Netherlands. The majority of patients presented with severe respiratory illness, but one case of AFM was seen in a 4-year-old boy (Knoester et al., 2017). Phylogenetic analysis of the samples revealed a different clustering to the 2014 outbreak strains. Simultaneously, similar upsurges were described by groups in Norway, Denmark, Germany, France, Spain, Portugal, Sweden, Wales (United Kingdom), Scotland (United Kingdom) and in the United States (Dyrdak et al., 2016; ECDC, 2016; Wang et al., 2017). In addition, cases of EV-D68 were also linked with AFM in Wales (2 cases), Scotland (5 cases), England (1 case), Sweden (3 cases), Italy (2 cases), Spain (3 cases), France (at least 1) and Argentina (15 cases) with strains clustering with a divergent B3 lineage (Antona et al., 2016; Dyrdak et al., 2016, ECDC, 2016; PAHO/WHO, 2016; Williams et al., 2016; Cabrerizo et al., 2017; Esposito et al., 2017; Giombini et al., 2017; Kirolos et al., 2017; Stacpoole et al., 2017). A subsequent surveillance study (Knoester et al., 2018) presented the results of a survey which found a total of 29 cases reported of EV-D68 infections associated with AFM in Europe in 2016. Interestingly, a higher number of EV-D68-associated AFM cases were reported in Europe in 2016 compared to 2014; 29 versus four cases in 2014. This could suggest the “new” circulating B3 subclade is more neuropathogenic or perhaps more transmissible than the B1 clade, which was most frequently reported in 2014. However, this most likely is due to increased surveillance and awareness established in 2014. In the United States, 149 AFM cases were reported in 2016, yet the exact number of AFM cases associated with EV-D68 is not known. Although there were fewer reported EV-D68 infections in 2016 in the United States overall, some AFM cases associated with EV-D68 were reported by Wang et al., 2017.

### Diagnosing an Enterovirus D68 Infection

#### Sample Collection

Depending on the clinical picture, several diagnostic samples can and should be collected to detect EV, such as CSF, feces, respiratory material and serum/plasma (Harvala et al., 2018). As most EV are transmitted via the fecal-oral route and replicate in the intestine, high viral loads are usually present in feces, therefore an effective material for detection and genotyping. Genotyping or serotyping are necessary and mandatory in cases of AFP to exclude poliovirus, and stool samples are essential to achieve this. However, EV-D68 is not readily detected in fecal samples, in addition it has a rhinovirus-like replication cycle in the nasal cavity. Indeed, the recently published PAHO/WHO report now recommends including a respiratory sample if AFP is suspected (PAHO/WHO report, 2017). Animal models have shown that EV-D68 can also disseminate to the CNS by retrograde axonal transport (Morrey et al., 2018). Multiple material types should therefore be collected if a patient presents with CNS symptoms: stool, CSF, blood and respiratory samples (Harvala et al., 2018).

#### Molecular Testing for Pan-Enterovirus Detection

Molecular testing is recognized as the gold standard for diagnosing an enterovirus infection. RT-PCR targeting the 5′UTR region has been established as a routine molecular test in many laboratories throughout Europe and other parts of the world (Harvala et al., 2018). It is used as a broad spectrum assay to detect the presence of EV, without regard to specific subtypes. The majority of assays are laboratory developed tests and can be used to screen a panel of suspected pathogens or to detect a specific target. Rapid and self-contained specimen-to-result tests such as the FilmArray system (BioFire/bioMerieux, Salt Lake City, United States) have also been used to screen and identify enterovirus /rhinovirus simultaneously by combining a nested multiplex PCR with melting curve analysis (Poritz et al., 2011). However, further tests would be needed as the Film-Array cannot distinguish enterovirus from rhinovirus. Additional FDA approved tests for enterovirus detection are the Cepheid GeneXpert and SmartCycler (Cepheid, Sunnyvale, California, United States). Finally, cell cultures can be used, however, they are not suitable for all enterovirus strains and further identification is required, using serotyping methods or real-time RT PCR.

A specific RT-PCR was developed to detect EV-D68 during a European surveillance project in 2014 (Poelman et al., 2015b). During an EV-D68 season (Summer-Autumn) or during an upsurge of the virus, a specific RT-PCR could be used for rapid diagnostics and patient management, and is particularly useful in the work-up of severe respiratory infections and AFM. During the 2014 outbreak, a study was carried out where the FilmArray system was used to detect positive enterovirus samples through its enterovirus/rhinovirus signals in a respiratory panel (Shibib et al., 2016). It was consequently found that a positive detection in the Rhinovirus 1 and 4 targets led to a high association (13.1× more likely) of EV-D68 found in subsequent samples sent to the CDC for confirmation. Further point of care tests were also evaluated during the outbreak. Similarly, the GenMark eSensor respiratory viral panel (Carlsbad, California, United States) was found to pick up low-positive rhinoviruses which were later found to be EV-D68 in 67% of samples, with a 94% sensitivity and 88% specificity rate (McAllister et al., 2015). This was subsequently noted due to cross-reactivity with rhinoviruses (McAllister et al., 2015; Diaz-Decaro et al., 2018).

#### Molecular Testing for Genotyping

Sanger sequencing of the VP1, and occasionally VP4-2 structural proteins, following detection of a positive EV sample is considered the gold standard for the determination of specific EV genotypes, according to World Health Organization (WHO) guidelines and Nix et al., 2006. Type specific RT-PCR and Sanger sequencing techniques have been used increasingly during outbreaks of EV-A71 in Asia and EV-D68 in the United States and Europe (Poelman et al., 2015a; Duong et al., 2016). Sequencing has transformed diagnostics, increasing the amount of knowledge on pathogens, their circulation and phylogeny. Phylogenetic analysis can be used more efficiently to look for genetic relationships within the EV subtype. However, typing enterovirus remains difficult due to vast variations in the genome (Midgley et al., 2015).

The development of these molecular tests has also allowed for the rapid detection and reporting of results in real time. A steady flow of epidemiological data should be available from health agencies or regional centers and fed back to the performing labs, and vice versa. This is not always optimal, and improvements could be achieved through for example the Antimicrobial, Infection and Prevention and Diagnostic stewardship model (Dik et al., 2016).

#### Next Generation Sequencing

Next generation sequencing, in comparison to the Sanger targeted approach, allows for the sequencing of multiple reads at once by reading optical signals after each base addition (Escalona et al., 2016). Useful information about changes in tropism or pathogenicity has been obtained by NGS. A retrospective study into the diversity of EV-D68 during the United States outbreak in 2014 using NGS technologies (metagenomic shotgun sequencing) revealed specific polymorphisms C3277A and A4020G, which triggered functional mutations at cleavage sites 2A^pro^ and 3C^pro^ respectively (Huang et al., 2015). These amino acid substitutions are suspected to alter protease activity and increase replication and transmission rates. The group also found similar mutations in a 2013 strain (US/CO/13-60), thought to be an ancestor of the 2014 outbreak strains. Indeed these coding polymorphisms were found to be present in poliovirus, as mentioned previously (Huang et al., 2015).

## Ev-D68 Association With Acute Flaccid Myelitis (AFM)

### The Strength of EV-D68 Association With AFM

**FIGURE 2:**
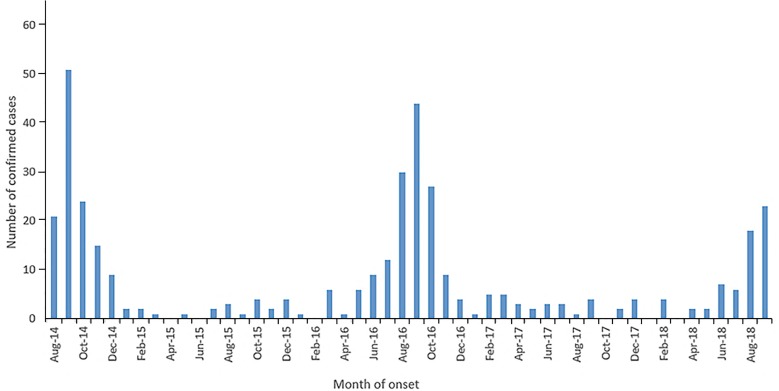
The number of confirmed United States AFM cases, published on the CDC by month of onset from August 2014-October 2018. The high number of confirmed AFM cases coincided with the 2014, 2016 and 2018 EV-D68 outbreaks. Figure taken from the NCIRD, AFM in the United States 2018. CDC and NCIRD (2018) retrieved from https://www.cdc.gov/acute-flaccid-myelitis/afm-surveillance.html.

The upsurge in infection and awareness has led to the expansion of current scientific knowledge, with several groups now investigating the depth of the association of EV-D68 with AFM. Many begin by describing the paralleling of numbers from the CDC for EV-D68 infections and the cases of AFM for 2014–2018 (Figure 2; Dyda et al., 2018; Messacar et al., 2018). From looking at the literature, during and after the United States 2014 outbreak, similar upsurges of severe respiratory and neurological symptoms were found worldwide, most notably in Northern Europe. The association was further strengthened by a group (Aliabadi et al., 2016) which found infection with EV-D68 resulted in a higher odds ratio than two control groups (10× and 4.5× respectfully) for AFM presentation. Furthermore, the respiratory prodromal phase prior to paralysis in 65% of patients (Martin et al., 2016), along with a high involvement of the cranial and spinal cord, appear to be more specifically associated with an EV-D68 infection (Messacar et al., 2018). Additionally, AFM mainly affects children, similarly to EV-D68, which indicates a specific target population (Dyda et al., 2018).

One of the challenges of the association is that EV-D68 is not always detected in clinical samples from AFM patients. One group (Greninger et al., 2015*)* found EV-D68 in respiratory samples in approximately 48% of AFM patients (25 cases), where no other pathogen including EV-D68, could be detected in CSF samples. Indeed, as discussed previously, detection of EV-D68 in CSF is uncommon. This has only been reported in a handful of cases (Kreuter et al., 2011; Esposito et al., 2016; Giombini et al., 2017; Ruggieri et al., 2017). A recent paper (Morrey et al., 2018) investigated this link through mouse models, deficient in interferon responses. Mice were challenged IP with EV-D68 of a known viral load. These researchers detected EV-D68 in the spinal cord just at early onset of paralysis, compared with the muscle injection site, which persisted for 6 weeks. The fact that the virus does not reside in the spinal cord for long could reflect the challenges in EV-D68 detection in CSF samples. Other viruses causing neurological disease including poliovirus, EV-A71, WNV and Rabies are similarly absent from CSF (Huang and Shih, 2015). Awareness of research in this area is particularly important for clinicians, as once an association is proven, they may be more inclined to take a respiratory sample and request for EV-D68 testing as well, during suspected AFM cases.

### Experiments Revealing the Neurotropic Nature of EV-D68

The unprecedented upsurge in AFM associated with EV-D68 has led to many questions into why this emerging virus has become so pathogenic, leading to the polio-like paralysis observed. At present the exact mechanism which EV-D68 uses to instigate infection remains largely unknown. Studies have shown that poliovirus can gain access to the CNS through axonal transport and neuromuscular junctions (Ohka et al., 1998; Huang and Shih, 2015), and it could be possible that EV-D68 follows a similar mechanism. A recent study in an EV-D68 mouse model, links paralysis to infections with EV-D68 through intramuscular injection by using various strains of the virus, including the 2014 outbreak strains. These injections resulted in the loss of motor neurons in the anterior horn of the corresponding spinal cord segments, leading to paralyzed limbs. The study goes on to suggest that replication of the virus in the motor neurons causes the damage, paralleling the development of neurological symptoms (Hixon et al., 2017b). Significantly, the study fulfills Koch’s postulates by activating paralysis in naïve mice from EV-D68 isolated from the spinal cord of a paralyzed mouse.

Much is still unknown about the exact mechanism EV-D68 uses to gain entry and replicate. Recently, a group investigating the life cycle of EV-D68 has started to answer some of the questions (Baggen et al., 2018). It was found that EV-D68 binds to synthetic glycoproteins through a terminally linked sialic acid. This binding induces a conformational change in the viral capsid, which commences the uncoating process, to inject the RNA and initiate the replication (Baggen et al., 2018). Sialic acid has been understood to be a binding site for other EV, such as Coxsackievirus A24, which was associated with acute haemorrhagic conjunctivitis (Baggen et al., 2018).

Wei et al. (2016) identified ICAM-5 as a possible entry receptor for EV-D68. EV-D68 was believed to bind to the ICAM-5-Fc receptor, where sialic acid was thought to induce a conformation change. Crucially, the telencephalon region of the brain was found to have enhanced ICAM-5 expression, this receptor could help explain the neurotropism of this virus (Wei et al., 2016; Messacar et al., 2018). Herpes Simplex Virus-1, another well-known neurotropic virus, also interacts with ICAM-5 to mediate cytokine secretion during infection (Tse et al., 2009). Although these studies shed a light on how EV-D68 may achieve its neuro-invasive capability, many more questions remain concerning the factors which determine the variability in disease severity which is seen in clinical cases. It is possible that changes in the genome, through mutational or selection pressure, led to an altered pathogenicity. Evidence in favor of this hypothesis has been shown by other authors, describing genogroup replacement between 2006 and 2014 (Xiang et al., 2016), and specific mutations in the puff region (key neutralization site) of VP2 in EV-D68 strains, which were isolated from patients with severe respiratory infections. Xiang et al. (2016) goes on to describe a mutational difference between the sequences obtained from the United States and China strains. In the United States strains there was a mutation in the pseudoknot structure in 3′-UTR, resulting in an altered phenotype comparatively to the Chinese strains. This could account for the differences in outbreaks between the United States and China in 2014.

### EV-D68 Case Definition

Although EV-D68 has shown similar neurological presentation to poliovirus, as well as similar MRI features, it has its own specific case definition. In response to the increased number of severe respiratory infections and number of acute paralysis cases during the 2014 outbreak the CDC proposed the case definition; “*onset of acute limb weakness on or after August 1, 2014, and a magnetic resonance image (MRI) showing a spinal cord lesion largely restricted to gray matter in a patient age ≤ 21 years*” (Washington State Department of Health, 2016). AFP patients who presented with pleocytosis (white blood cells >5 mm^3^) in their CSF who had a negative or no MRI result were recommended as a probable case. The terms AFM and AFP are used interchangeably in articles and reports from 2014. Adding to the confusion is the restriction of the AFM case definition to individuals younger than 21 years of age, as it is currently understood that AFM does occur in adults as well. Although mostly children with a chronic illness were affected, children and adults without any known underlying condition were also reported (Williams et al., 2016; Stacpoole et al., 2017). Additionally, it must be updated in accordance with increased data gained from reports.

The establishment of a case definition for AFM was particularly important as other diseases such as GBS, may also present with AFP. Although, paralysis tends to be more symmetrical in GBS (Jasti et al., 2016). Cases of EV-D68 associated AFM could be diagnosed as “atypical-GBS,” unless an MRI scan is made or electromyography examinations are carried out. Typically, EV-D68 associated AFM develops following acute febrile respiratory syndrome, up to 2 weeks prior to onset of weakness (Tyler, 2015). Prodromal symptoms compatible with respiratory infections including shortness of breath (82%), cough (82%), and rhinorrhoea or nasal congestion (71%) (Martin et al., 2016) could be incorporated into the case definition.

### Clinical Characteristics and Diagnosis of EV-D68 AFM

**FIGURE 3:**
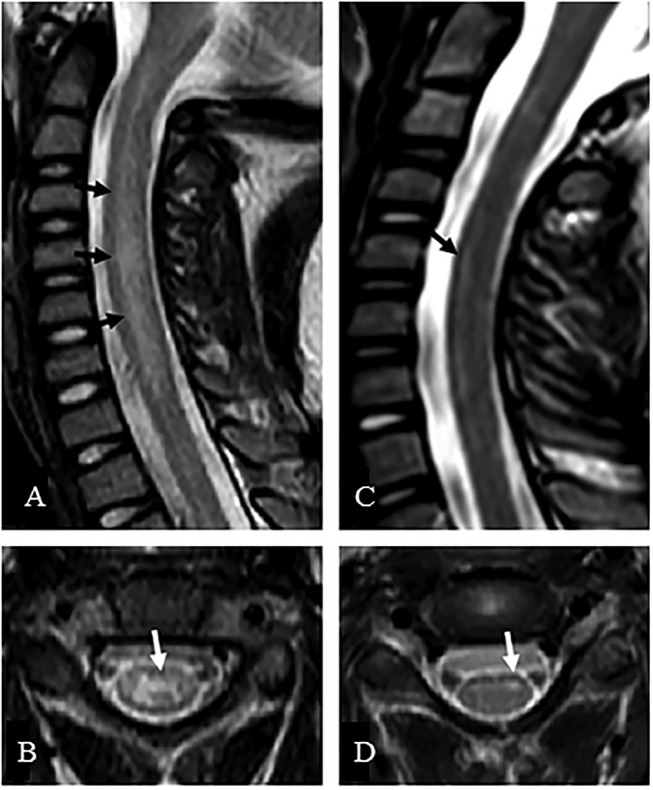
MRI of a suspected EV-D68 AFM patient. The MRI presents sagittal **(A,C)** and axial images **(B,D)** of the central nerves system. **(A)** Presents a case where the whole central gray matter was involved, producing a characteristic “H” pattern on axial image **(B)**. **(C)** Presents a case where T2 hyperintensity was confined to the left anterior horn cells, which is demonstrated on the axial image **(D)**. Taken from Maloney et al., 2015. Order License Id: 4382500446364.

**FIGURE 4:**
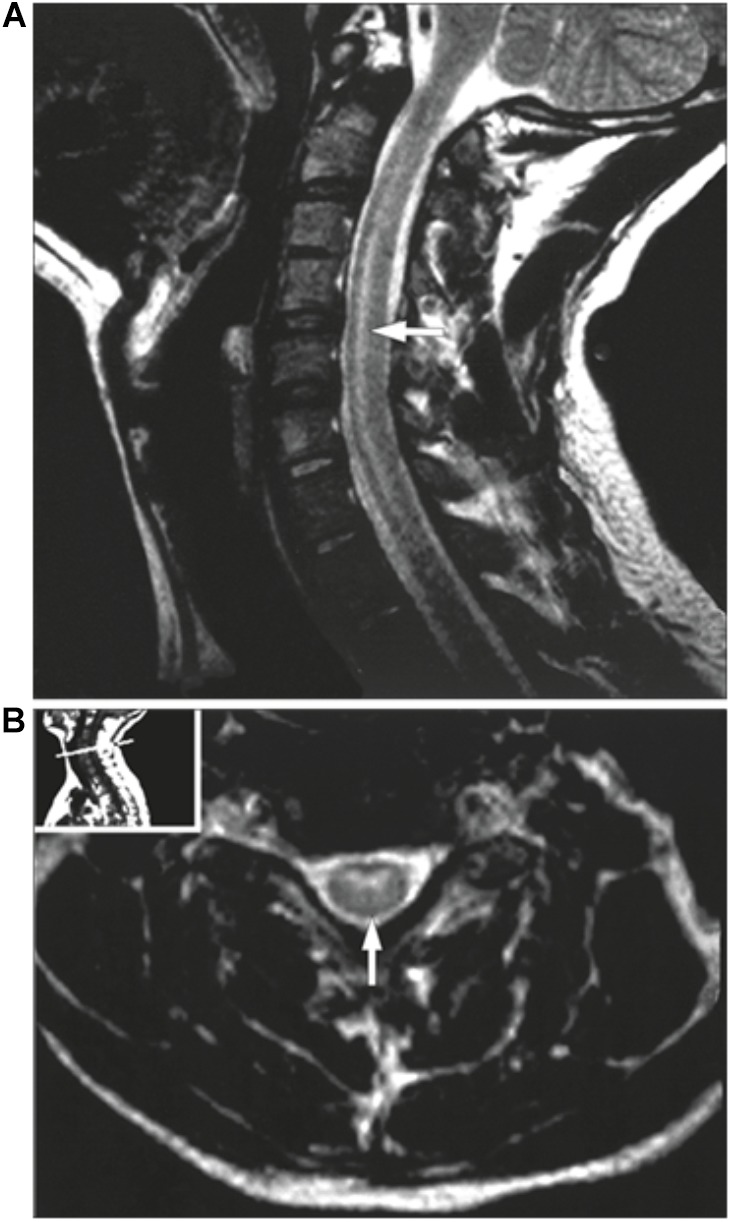
MRI of a poliovirus AFM patient. The MRI presents a sagittal **(A)** and an axial image **(B)** of the central nerves system. **(A)** presents a case showing hyperintensities involving the anterior horn cells from C3 to C7. **(B)** demonstrates the same case as an axial image. Taken from Haq and Wasay (2006). Order License Id: 4382500944212.

Patients presenting with a suspected EV-D68 associated AFM should undergo a series of examinations to confirm this diagnosis. One of the most valuable examinations, MRI, was used extensively during the 2014 outbreak to facilitate the establishment of a case definition. Patients can present with a variety of symptoms varying from cough, runny nose and diarrhea to muscles aches, fever and in some cases respiratory distress, particularly in children younger than five (National Center for Immunization and Respiratory Diseases [NCIRD], Division of Viral Diseases, 2017; Knoester et al., 2018). AFM symptoms are typically described as asymmetric motor weakness mostly affecting the upper limbs in the majority of current known cases. The weakness is flaccid, with deep-tendon reflexes reduced or absent (CDC and NCIRD, 2015). The cranial nerves are commonly affected with symptoms such as facial weakness, dysarthria and dysphagia being described (Tyler, 2015). Most patients did undergo a spinal tap on presentation, which showed cerebrospinal fluid pleocytosis in the majority of cases (Tyler, 2015). An MRI scan is essential for the diagnosis, as it shows lesions in the anterior horn of the gray matter along the spinal cord and sometimes in the brainstem (Tyler, 2015). As shown in the MRI images (Figures 3,4), these distinctive lesions point at the involvement of the spinal cord motor neurons. It must be noted that the lesions seen on the MRI scans during cases of EV-D68 associated AFM are identical to lesions in the spinal cord which are found in poliomyelitis. Diagnosing a case of AFM requires input from radiologists, clinical virologists, pediatricians and neurologists and highlights the need for communication between specialists to ensure a case is recognized, with the appropriate samples and tests requested, including PCR and MRI scans.

### Treatment Options

Increasing reports of enhanced pathogenicity and the severity of the paralysis in the affected individuals have led to urgency in finding an effective treatment for EV-D68. At present, there is no vaccine or therapy which exists against EV-D68. A study carried out by Rhoden et al. (2015) evaluated several anti-viral candidates for EV-D68, with some promising results; V-7404, a protease inhibitor currently in development to treat poliovirus in immunodeficient patients; DAS181, similarly in development however to treat Influenza and Parainfluenza virus infections, and finally Rupintrivir, another protease inhibitor, not currently being developed further. Both V-7404 and Rupintrivir were able to inhibit all four tested EV-D68 strains (one Fermon and three 2014 outbreak strains). DAS181, which works as a sialidase was comparable in effectiveness, with a slightly EC50. However, these results were only obtained from *in vitro* testing, and clinical studies with these agents will not be available in the intermediate future.

A further study (Tyler, 2015) investigated the use of an already FDA approved drug, Fluoxetine, a serotonin inhibitor normally used as an antidepressant. The study found Fluoxetine inhibited the replication of EV-D68 in HeLa cells by a direct interaction with the 2C protein, which is thought to have a function in assembly. It is unknown if the tested strain was from the 2014 outbreak. However, Fluoxetine administration was noted to result in low maximal plasma levels, which could lead to problems in *in vivo* investigations (Rhoden et al., 2015).

As EV-D68 is a relatively emerging pathogen in terms of increased pathogenicity in recent years, treatment options are still far off. Exploring existing treatments for viruses, other than EV-D68, which present with similar symptoms, is therefore an attractive strategy (CDC and NCIRD, 2014). Recently, investigations were carried out (Hixon et al., 2017a) into three different empiric therapies that could decrease the severity of paralysis in a mouse model. The mice were intramuscularly injected with one of the 2014 outbreak strains. Positive results were obtained with hIVIG, which reduced paralysis and spinal cord viral loads. Fluoxetine was shown to have a neutral effect, contrasting to Tyler, 2015 and dexamethasone worsened outcomes for the mice, causing increased mortality, possibly due to reducing the immune response and increasing viral replication. Disadvantageous results of corticosteroid treatment were also seen when these agents were used in an outbreak of neuro-invasive EV-A71 in Cambodia (World Health Organization [WHO], 2012). So far this research presents promising results for hIVIG therapy (pre and early post-infection) by providing potential protection against one of the most severe manifestations of an EV-D68 infection. Indeed, hIVIG therapy has been used previously and has arguably been successful in a least one suspected AFM case associated with WNV (Walid and Mahmoud, 2009). However, a further study (Chong et al., 2018) found hIVIG given to patients initially after onset of neurological symptoms, led to a poor prognosis.

As permanent functional impairment appears to be common in EV-D68 associated AFM (Messacar et al., 2016), solutions are also being sought to improve the outcome of patients after the paralysis has become irreversible. A potentially promising technique is nerve and muscle transfer, which was historically used for poliomyelitis associated paralysis (Messacar et al., 2016). As such, one study (Saltzman et al., 2016) describes the results of a trial involving nerve transfers in several patients following AFM associated EV-D68. In most of the cases proximal nerves were transferred, with one case undergoing bilateral nerve transfers. The study showed promising results with some muscle strength regained over a 6-month follow-up.

### Non-polio Enterovirus Awareness and Its Subsequent Increased Surveillance

In the United States, published results from various surveillance studies are presented through the CDC. However, EV-D68 is only voluntarily reported in most states in the United States and is not a national notifiable disease (CDC and NNDSS, 2018). Similarly, the ECDC does not have an active surveillance system in place and relies on member states to provide updates on circulation of various EV. Therefore non-polio enterovirus surveillance in Europe can also depend on local interests of specific laboratories and national health institutes. As it is not mandatory to report EV-D68 in Europe (except Norway), and just in a few states in the United States, little real-time information is known on the exact numbers. This is a current challenge for EV-D68 data. However this may change now that the PAHO/WHO has introduced the recommendation of including respiratory samples in suspected EV-D68 cases (PAHO/WHO report, 2017).

Several surveillance systems are in place throughout Europe. One example is the enhanced non-polio EV surveillance system implemented in Denmark in which respiratory samples were included, along with clinical description and genetic characterisation of the viruses (Barnadas et al., 2017). Similarly, in collaboration with the Dutch National Institute of Health, the TYPENED initiative [Typing network Netherlands] is involved in sequencing and collection of data (Niesters et al., 2013). In France, two EV National Reference Laboratories (in Clermont-Ferrand and Lyon) report the number of enterovirus infections and type of samples analyzed on a regular basis.^1^ (Schuffenecker et al., 2016). Additionally, networks such as ENPEN exist to increase knowledge and communicate both epidemiological and clinical data during outbreaks, and to chart emerging infections. Such networks have been instrumental in raising awareness of EV-D68 and other non-polio EV through an email alert system and conferences (Harvala et al., 2018).

## Discussion

### Burden of Disease and the Problems of Underdiagnosing

Enterovirus D68 made headlines worldwide in 2014 as a mysterious, relatively unknown virus was capable of causing severe illness. The CDC currently monitors and provides monthly updates on the incidence of AFM, which subsequently revealed another upsurge in 2016. Since no recent data has been published on the number of confirmed EV-D68 infections or the number of AFM cases in Europe, more testing and communication is required to understand both the EV-D68 infection patterns and the frequency at which this virus causes AFM. Specific EV-D68 assays have been developed to rapidly diagnose infections. Despite the existing evidence for the association between EV-D68 and AFM (Hixon et al., 2017b; Messacar et al., 2018), EV-D68 is overlooked due to insufficient knowledge, sampling, laboratory testing and communication between healthcare professionals and little surveillance from public health authorities.

### Future Perspective and Directions

The current surveillance systems for poliovirus such as GPEI, environmental and AFP surveillance have been instrumental in nearly eradicating polio. Integrating EV-D68 into these established surveillance systems, would be highly effective in understanding the true burden of disease and prepare hospitals and laboratories for upcoming outbreaks. NGS has the potential to be a powerful tool in investigating emerging and untypeable pathogens. It could also be used to understand the host-pathogen relationship during an infection and to understand the evolution of these viruses. However, there are still both technical and financial obstacles left to overcome before its used in routine practice (Rutvisuttinunt et al., 2017).

Further outbreaks of EV-D68 can be expected and could subsequently lead to an increase in EV-D68 associated AFM cases. EV-D68 numbers and AFM cases have increased in autumn of 2018. This has been communicated by the EV-D68 network made up of virologists and clinicians who collaborated during the 2014 and 2016 outbreaks. This has included two recent AFM cases from the Netherlands. Preliminary typing results obtained from the University Medical Center Groningen, the Netherlands, have indicated that recent EV-D68 samples have clustered into the B3 and A2 subclades. It is imperative to have effective and streamlined diagnostic procedures along with stewardship models to deal with the potential increase in cases. As a result, awareness needs to be created targeting clinicians and hospital wards in order to make clinical staff are aware of the virus. Continued interdisciplinary communication is important to ensure EV-D68 is translated across each medical field appropriately.

As effective treatment of EV-D68 infections and AFM are thus far unsubstantial, the focus may need to be placed on the development of a vaccine. Currently efficient vaccines are available for a few members of the enterovirus genus i.e., poliovirus and EV-A71 (Klein and Chong, 2015). These viruses however, have shown high incidences therefore it could be questioned whether a vaccine should be developed for EV-D68 at this time for economic reasons. Yet these vaccines have transformed the fight against these fatal EV. Using current knowledge and approaches, development of a vaccine against EV-D68 is technically achievable. A group recently published the results of a trial involving insect cell-expressed EV-D68 virus-like particle as a promising candidate for an EV-D68 vaccine (Dai et al., 2018). At present, the number of infections and life-threating cases do not reflect those of poliovirus, possibly due to lack of reporting with the current voluntary reporting systems. Only increased surveillance and diagnosis will make it possible to expose the extent of the EV-D68 threat.

## Author Contributions

HC drafted the manuscript. RP, MK, CVL-B, and HN revised the work critically and gave final approval before publication.

## Conflict of Interest Statement

The authors declare that the research was conducted in the absence of any commercial or financial relationships that could be construed as a potential conflict of interest.

## Footnotes

1 http://cnr.chu-clermontferrand.fr/CNR/

## Abbreviations

AFM: acute flaccid myelitis
AFP: acute flaccid paralysis
CDC: Centers for Disease Control and Prevention
CNS: central nerve system
CSF: cerebral spinal fluid
EC50: lower effective concentration
ECDC: European Centre for Disease Prevention and Control
ENPEN: European Non-Polio Enterovirus Network
ESCV: European Society of Clinical Virology
EV: enteroviruses
EV-A71: enterovirus A71
EV-D68: enterovirus D68
FDA: Food and Drug Administration
GBS: Guillain-Barré syndrome
GPEI: Global Polio Eradication Initiative
hIVIG: human intravenous immunoglobulin
ICAM-5: neuron-expressed intercellular adhesion molecule 5
IP: intraperitoneally
MRI: magnetic resonance imaging
NCIRD: The National Center for Immunization and Respiratory Diseases
NGS: next generation sequencing
NNDSS: National Notifiable Diseases Surveillance System
PAHO: Pan American Health Organization
RNA: ribonucleic acid
RT-PCR: reverse transcriptase PCR
USA: United States
UTR: untranslated region
VP1: viral protein 1
VP4-2: Complete viral protein 4 and partial viral protein 2
WHO: World Health Organization
WNV: West Nile virus

## References

[B1] Aliabadi N. Messacar K. Pastula D. Robinson C. Leshem E. Sejvar J. (2016). Enterovirus D68 infection in children with acute flaccid Myelitis, Colorado, USA, 2014. *Emerg. Infect. Dis.* 22 1387–1394. 10.3201/eid2208.151949 27434186 PMC4982171

[B2] Antona D. Kossorotoff M. Schuffenecker I. Mirand A. Leruez-Ville M. Bassi C. (2016). Severe paediatric conditions linked with EV-A71 and EV-D68, France, May to October 2016. *Euro Surveill.* 21:30402. 10.2807/1560-7917.ES.2016.21.46.30402 27918268 PMC5144948

[B3] Baggen J. Thibaut H. Strating J. Van Kuppeveld F. (2018). The life cycle of non-polio enteroviruses and how to target it. *Nat. Rev. Microbiol.* 16 368–381. 10.1038/s41579-018-0005-4 29626210

[B4] Barnadas C. Midgley S. Skov M. Jensen L. Poulsen M. Fischer T. (2017). An enhanced *Enterovirus* surveillance system allows identification and characterization of rare and emerging respiratory enteroviruses in Denmark, 2015– 16. *J. Clin. Virol.* 93 40–44. 10.1016/j.jcv.2017.05.017 28618288

[B5] Blomqvist S. Savolainen C. Råman L. Roivainen M. Hovi T. (2002). Human rhinovirus 87 and enterovirus 68 represent a unique serotype with rhinovirus and enterovirus features. *J. Clin. Microbiol.* 40 4218–4223. 10.1128/JCM.40.11.4218-4223.2002 12409401 PMC139630

[B6] Cabrerizo M. García-Iñiguez J. P. Munell F. Madurga-Revilla P. Rodrigo C. Martínez-Sapiña A. (2017). First cases of severe flaccid paralysis associated with enterovirus D68 infection in Spain, 2015–2016. *Pediatr. Infect. Dis. J.* 36 1214–1216. 10.1097/INF.0000000000001668 28661963

[B7] CDC and NCIRD (2018). *Acute Flaccid Myelitis Investigation.* Available at: https://www.cdc.gov/acute-flaccid-myelitis/afm-surveillance.html [accessed July 03, 2018]

[B8] CDC and NCIRD (2014). *Acute Flaccid Myelitis: Interim Considerations for Clinical Management.* Available at: https://www.cdc.gov/acute-flaccid-myelitis/hcp/clinical-management.html [accessed July 03, 2018]

[B9] CDC and NCIRD (2015). *For Clinicians: Diagnosing Acute Flaccid Myelitis (AFM) in the United States.* Available at: https://www.cdc.gov/acute-flaccid-myelitis/downloads/afm-presentation.pdf [accessed July 03, 2018]

[B10] CDC and NNDSS (2018). *National Notifiable Conditions.* Available at: https://wwwn.cdc.gov/nndss/conditions/notifiable/2018/ [accessed April 16, 2018].

[B11] Chong P. Kira R. Mori H. Okumura A. Torisu H. Yasumoto S. (2018). Clinical features of acute flaccid myelitis temporally associated with an enterovirus D68 outbreak: results of a nationwide survey of acute flaccid paralysis in Japan, August-December 2015. *Clin. Infect. Dis.* 66 653–664. 10.1093/cid/cix860 29028962 PMC5850449

[B12] Craighead J. (ed.) (2000). “Enteroviruses,” in *Pathology and Pathogenesis of Human Viral Disease* (Amsterdam: Elsevier) 1–28. 10.1016/B978-012195160-3/50002-9

[B13] Dai W. Zhang C. Zhang X. Xiong P. Liu Q. Gong S. (2018). A virus-like particle vaccine confers protection against enterovirus D68 lethal challenge in mice. *Vaccine* 36 653–659. 10.1016/j.vaccine.2017.12.057 29295756

[B14] Diaz-Decaro J. Green N. Godwin H. (2018). Critical evaluation of FDA-approved respiratory multiplex assays for public health surveillance. *Expert Rev. Mol. Diagn.* 18 631–643. 10.1080/14737159.2018.1487294 29886764 PMC7103694

[B15] Dik J.-W. Poelman R. Friedrich A. Panday P. Lo-Ten-Foe J. Van Assen S. (2016). An integrated stewardship model: antimicrobial, infection prevention and diagnostic (AID). *Future Microbiol.* 11 93–102. 10.2217/fmb.15.99 26323589

[B16] Duong V. Mey C. Eloit M. Zhu H. Danet L. Huang Z. (2016). Molecular epidemiology of human enterovirus 71 at the origin of an epidemic of fatal hand, foot and mouth disease cases in Cambodia. *Emerg. Microbes. Infect.* 5:e104. 10.1038/emi.2016.101 27651091 PMC5113052

[B17] Dyda A. Stelzer-Braid S. Adam D. Chughtai A. Raina MacIntyre C. Amalie D. (2018). The association between acute flaccid myelitis (AFM) and *Enterovirus* D68 (EV-D68) – what is the evidence for causation? *Euro Surveill.* 23:17-00310. 10.2807/1560-7917.ES.2018.23.3.17-00310 29386095 PMC5792700

[B18] Dyrdak R. Grabbe M. Hammas B. Ekwall J. Hansson K. E. Luthander J. (2016). Outbreak of enterovirus D68 of the new B3 lineage in Stockholm, Sweden, August to September 2016. *Euro Surveill.* 21:30403. 10.2807/1560-7917.ES.2016.21.46.30403 27918255 PMC5144949

[B19] ECDC. (2016). *Rapid Risk Assessment – Enterovirus Detections Associated with Severe Neurological Symptoms in Children and Adults in European Countries.* Solna Municipality: ECDC.

[B20] Escalona M. Rocha S. Posada D. (2016). A comparison of tools for the simulation of genomic next-generation sequencing data. *Nat. Rev. Genet.* 17 459–469. 10.1038/nrg.2016.57 27320129 PMC5224698

[B21] Esposito S. Bosis S. Niesters H. Principi N. (2015). Enterovirus D68 infection. *Viruses* 7 6043–6050. 10.3390/v7112925 26610548 PMC4664996

[B22] Esposito S. Chidini G. Cinnante C. Napolitano L. Giannini A. Terranova L. (2017). Acute flaccid myelitis associated with enterovirus-D68 infection in an otherwise healthy child. *Virol. J.* 14:4. 10.1186/s12985-016-0678-0 28081720 PMC5234096

[B23] Esposito S. Lunghi G. Zampiero A. Tagliabue C. Orlandi A. Torresani E. (2016). Enterovirus-D68 in the cerebrospinal fluid of two children with aseptic meningitis. *Pediatr. Infect. Dis. J.* 35 589–591. 10.1097/INF.0000000000001085 26859634

[B24] Foster C. B. Friedman N. Carl J. Piedimonte G. (2015). Enterovirus D68: a clinically important respiratory enterovirus. *Cleve. Clin. J. Med.* 82 26–31. 10.3949/ccjm.82a.14166 25552624

[B25] Giombini E. Rueca M. Barberi W. Iori A. P. Castilletti C. Scognamiglio P. (2017). Enterovirus D68–associated acute flaccid myeltis in immunocompromised women in Italy. *Emerg. Infect. Dis.* 23 1690–1693. 10.3201/eid2310.170792 28930024 PMC5621549

[B26] Gong Y.-N. Yang S.-L. Shih S.-R. Huang Y.-C. Chang P.-Y. Huang C.-G. (2016). Molecular evolution and the global reemergence of enterovirus D68 by genome-wide analysis. *Medicine* 95:e4416. 10.1097/MD.0000000000004416 27495059 PMC4979813

[B27] Greninger A. Naccache S. Messacar K. Clayton A. Yu G. Somasekar S. (2015). A novel outbreak enterovirus D68 strain associated with acute flaccid myelitis cases in the USA (2012-14): a retrospective cohort study. *Lancet Infect. Dis.* 15 671–682. 10.1016/S1473-3099(15)70093-9 25837569 PMC6027625

[B28] Haq A. Wasay M. (2006). Magnetic resonance imaging in poliomyelitis. *JAMA Neurol.* 63:778. 10.1001/archneur.63.5.778 16682551

[B29] Harvala H. Broberg E. Benschop K. Berginc N. Ladhani S. Susi P. (2018). Recommendations for enterovirus diagnostics and characterisation within and beyond Europe. *J. Clin. Virol.* 101 11–17. 10.1016/j.jcv.2018.01.008 29414181

[B30] Hixon A. Clarke P. Tyler K. (2017a). Evaluating treatment efficacy in a mouse model of enterovirus D68-associated paralytic myelitis. *J. Infect. Dis.* 216 1245–1253. 10.1093/infdis/jix468 28968718 PMC5853295

[B31] Hixon A. Yu G. Leser J. Yagi S. Clarke P. Chiu C. (2017b). A mouse model of paralytic myelitis caused by enterovirus D68. *PLoS Pathogens* 13:e1006199. 10.1371/journal.ppat.1006199 28231269 PMC5322875

[B32] Holm-Hansen C. Midgley S. Fischer T. (2016). Global emergence of enterovirus D68: a systematic review. *Lancet Infect. Dis.* 16 e64–e75. 10.1016/S1473-3099(15)00543-5 26929196

[B33] Huang H. Shih S. (2015). Neurotropic enterovirus infections in the central nervous system. *Viruses* 7 6051–6066. 10.3390/v7112920 26610549 PMC4664993

[B34] Huang W. Wang G. Zhuge J. Nolan S. Dimitrova N. Fallon J. (2015). Whole-Genome sequence analysis reveals the enterovirus D68 isolates during the United States 2014 outbreak mainly belong to a novel clade. *Sci. Rep.* 5:15223. 10.1038/srep15223 26469882 PMC4606740

[B35] Imamura T. Okamoto M. Nakakita S.-I. Suzuki A. Saito M. Tamaki R. (2014). Antigenic and receptor binding properties of enterovirus. *J. Virol.* 88 2374–2384. 10.1128/JVI.03070-13 24371050 PMC3958110

[B36] Imamura T. Oshitani H. (2015). Global reemergence of enterovirus D68 as an important pathogen for acute respiratory infections. *Rev. Med. Virol.* 25 102–114. 10.1002/rmv.1820 25471236 PMC4407910

[B37] Jasti A. Selmi C. Sarmiento-Monroy J. Vega D. Anaya J. Gershwin M. (2016). Guillain-Barré syndrome: causes, immunopathogenic mechanisms and treatment. *Expert Rev. Clin. Immunol.* 12 1175–1189. 10.1080/1744666X.2016.1193006 27292311

[B38] Kaida A. Iritani N. Yamamoto S. Kanbayashi D. Hirai Y. Togawa M. (2017). Distinct genetic clades of enterovirus D68 detected in 2010 2013 and 2015 in Osaka City, Japan. *PLoS One* 12:e0184335. 10.1371/journal.pone.0184335 28902862 PMC5597212

[B39] Kirolos A. Mark K. Waugh C. Shetty J. McCallum A. Templeton K. (2017). Cluster of acute flaccid paralysis in children following enterovirus D68 infection in Scotland. *Eur. J. Public Health.* 27:ckx187.697 10.1093/eurpub/ckx187.697

[B40] Klein M. Chong P. (2015). Is a multivalent hand, foot, and mouth disease vaccine feasible? *Hum. Vaccin. Immunother*. 11 2688–2704. 10.1080/21645515.2015.1049780 26009802 PMC4685682

[B41] Knoester M. Helfferich J. Poelman R. Van Leer-Buter C. Brouwer O. Niesters H. (2018). Twenty-Nine cases of enterovirus-D68 associated acute flaccid myelitis in Europe 2016; A case series and epidemiologic overview. *Pediatr. Infect. Dis. J.* 10.1097/INF.0000000000002188 [Epub ahead of print]. 30234793 PMC6296836

[B42] Knoester M. Schölvinck E. Poelman R. Smit S. Vermont C. Niesters H. (2017). Upsurge of enterovirus D68 the Netherlands, 2016. *Emerg. Infect. Dis.* 23 140–143. 10.3201/eid2301.161313 27660916 PMC5176244

[B43] Kreuter J. D. Barnes A. McCarthy J. E. Schwartzman J. D. Oberste M. S. Rhodes C. H. (2011). A fatal central nervous system enterovirus 68 infection. *Arch. Pathol. Lab. Med.* 135 793–796. 10.1043/2010-0174-CR.1 21631275

[B44] Lang M. Mirand A. Savy N. Henquell C. Maridet S. Perignon R. (2014). Acute flaccid paralysis following enterovirus D68 associated pneumonia, France, 2014. *Euro Surveill.* 19:20952 10.2807/1560-7917.ES2014.19.44.20952 25394254

[B45] Levy A. Roberts J. Lang J. Tempone S. Kesson A. Dofai A. (2015). Enterovirus D68 disease and molecular epidemiology in Australia. *J. Clin. Virol.* 69 117–121. 10.1016/j.jcv.2015.06.079 26209392

[B46] Maloney J. Mirsky D. Messacar K. Dominguez S. Schreiner T. Stence N. (2015). MRI findings in children with acute flaccid paralysis and cranial nerve dysfunction occurring during the 2014 enterovirus D68 outbreak. *Am. J. Neuroradiol.* 36 245–250. 10.3174/ajnr.A4188 25414005 PMC7965662

[B47] Martin G. Li R. Cook V. Carwana M. Tilley P. Sauve L. (2016). Respiratory presentation of pediatric patients in the 2014 enterovirus D68 outbreak. *Can. Respir. J.* 2016:8302179. 10.1155/2016/8302179 27610028 PMC5004002

[B48] McAllister S. Schleiss M. Arbefeville S. Steiner M. Hanson R. Pollock C. (2015). Epidemic 2014 enterovirus D68 cross-reacts with human rhinovirus on a respiratory molecular diagnostic platform. *PLoS One* 10:e0118529. 10.1371/journal.pone.0118529 25799541 PMC4370466

[B49] Messacar K. Asturias E. Hixon A. Van Leer-Buter C. M. Niesters H. Tyler K. (2018). Personal view enterovirus D68 and acute flaccid myelitis—evaluating the evidence for causality. *Lancet Infect. Dis.* 18 e239–e247. 10.1016/S1473-3099(18)30094-X 29482893 PMC6778404

[B50] Messacar K. Schreiner T. Van Haren K. Yang M. Glaser C. Tyler K. (2016). Acute flaccid myelitis: a clinical review of US cases 2012–2015. *Ann. Neurol.* 80 326–338. 10.1002/ana.24730 27422805 PMC5098271

[B51] Midgley C. Watson J. Nix W. Curns A. Rogers S. Brown B. (2015). Severe respiratory illness associated with a nationwide outbreak of enterovirus D68 in the USA (2014): a descriptive epidemiological investigation. *Lancet Respir. Med.* 3 879–887. 10.1016/S2213-2600(15)00335-5 26482320 PMC5693332

[B52] Morrey J. Wang H. Hurst B. Zukor K. Siddharthan V. Van Wettere A. (2018). Causation of acute flaccid paralysis by myelitis and myositis in enterovirus-D68 infected mice deficient in interferon αβ/γ receptor deficient mice. *Viruses* 10:E33. 10.3390/v10010033 29329211 PMC5795446

[B53] National Center for Immunization and Respiratory Diseases [Ncird], Division of Viral Diseases (2017). *Enterovirus D68.* Available at: https://www.cdc.gov/non-polio-enterovirus/about/ev-d68.html [accessed April 11, 2018]

[B54] Niesters H. Rossen J. van der Avoort H. Baas D. Benschop K. Claas E. (2013). Laboratory-based surveillance in the molecular era: the TYPENED model, a joint data-sharing platform for clinical and public health laboratories. *Euro Surveill.* 18:20387. 10.2807/ese.18.04.20387-en 23369392

[B55] Nix A. W. Oberste M. Pallansch M. (2006). Sensitive, seminested PCR amplification of VP1 sequences for direct identification of all enterovirus serotypes from original clinical specimens. *J. Clin. Microbiol.* 44 2698–2704. 10.1128/JCM.00542-06 16891480 PMC1594621

[B56] Noor A. Krilov L. (2016). Enterovirus infections. *Pediatr. Rev.* 37 505–515. 10.1542/pir.2016-0103 27909105

[B57] Ohka S. Yang W.-X. Terada E. Iwasaki K. Nomoto A. (1998). Retrograde transport of intact poliovirus through the axon via the fast transport system. *Virology* 250 67–75. 10.1006/viro.1998.9360 9770421

[B58] PAHO/WHO. (2016). *Report From: The Argentina International Health Regulations National Focal Point.* Washington, DC: PAHO.

[B59] PAHO/WHO report. (2017). *Epidemiological Alert Acute Flaccid Myelitis Associated with Enterovirus D68 in the Context of Acute Flaccid Paralysis Surveillance.* Washington, DC: PAHO.

[B60] Paul J. (1971). *A History of Poliomyelitis.* New Haven, CT: Yale University Press.

[B61] Pfeiffer H. Bragstad K. Skram M. Dahl H. Knudsen P. Chawla M. (2015). Two cases of acute severe flaccid myelitis associated with enterovirus D68 infection in children, Norway, autumn 2014. *Euro Surveill.* 20:21062. 10.2807/1560-7917.ES2015.20.10.21062 25788251

[B62] Poelman R. Schölvinck E. Borger R. Niesters H. Van Leer-Buter C. (2015a). The emergence of enterovirus D68 in a Dutch University Medical Center and the necessity for routinely screening for respiratory viruses. *J. Clin. Virol.* 62 1–5. 10.1016/j.jcv.2014.11.011 25542461 PMC7185662

[B63] Poelman R. Schuffenecker I. Van Leer-Buter C. Josset L. Niesters H. Lina B. (2015b). European surveillance for enterovirus D68 during the emerging North-American outbreak in 2014. *J. Clin. Virol.* 71 1–9. 10.1016/j.jcv.2015.07.296 26364237

[B64] Poritz M. Blaschke A. Byington C. Meyers L. Nilsson K. Jones D. (2011). Filmarray, an automated nested multiplex PCR system for multi-pathogen detection: development and application to respiratory tract infection. *PLoS One* 6:e26047. 10.1371/journal.pone.0026047 22039434 PMC3198457

[B65] Rahamat-Langendoen J. Riezebos-Brilman A. Borger R. van der Heide R. Brandenburg A. Schölvinck E. (2011). Upsurge of human enterovirus 68 infections in patients with severe respiratory tract infections. *J. Clin. Virol.* 52 103–106. 10.1016/j.jcv.2011.06.019 21802981

[B66] Rhoden E. Zhang M. Nix W. Oberste M. (2015). In vitro efficacy of antiviral compounds against enterovirus D68. *Antimicrob. Agents Chemother.* 59 7779–7781. 10.1128/AAC.00766-15 26149998 PMC4649146

[B67] Ruggieri V. Paz M. I. Peretti M. G. Rugilo C. Bologna R. Freire C. (2017). Enterovirus D68 infection in a cluster of children with acute flaccid myelitis, Buenos Aires, Argentina, 2016. *Eur. J. Paediatr. Neurol.* 21 884–890. 10.1016/j.ejpn.2017.07.008 28747261

[B68] Rutvisuttinunt W. Klungthong C. Thaisomboonsuk B. Chinnawirotpisan P. Ajariyakhajorn C. Manasatienkij W. (2017). Retrospective use of next-generation sequencing reveals the presence of Enteroviruses in acute influenza-like illness respiratory samples collected in South/South-East Asia during 2010–2013. *J. Clin. Virol.* 94 91–99. 10.1016/j.jcv.2017.07.004 28779659 PMC7106496

[B69] Saltzman E. Vu A. Schwenker A. Raney S. Sneag D. Yakuboff K. (2016). Nerve transfers for enterovirus D68-induced acute flaccid myelitis. *HAND* 11:97S. 10.1016/j.pediatrneurol.2018.07.018 30301588

[B70] Schuffenecker I. Mirand A. Josset L. Henquell C. Hecquet D. Pilorgé L. (2016). Epidemiological and clinical characteristics of patients infected with enterovirus D68 France, July to December 2014. *Euro Surveill.* 21:30226. 10.2807/1560-7917.ES.2016.21.19.30226 27195770

[B71] Sejvar J. Lopez A. Cortese M. Leshem E. Pastula D. Miller L. (2016). Acute flaccid myelitis in the United States, August-December 2014: results of nationwide surveillance. *Clin. Infect. Dis.* 63 737–745. 10.1093/cid/ciw372 27318332 PMC5709818

[B72] Shibib D. Matushek S. Beavis K. Gawel S. Charnot-Katsikas A. (2016). BioFire film array respiratory panel for detection of enterovirus D68. *J. Clin. Microbiol.* 54 457–459. 10.1128/JCM.02339-15 26607982 PMC4733204

[B73] Stacpoole S. Molyneux A. Bäumer D. (2017). Acute segmental poliomyelitis-like flaccid paralysis in an adult in the UK, associated with enterovirus D68. *Pract. Neurol.* 17 297–301. 10.1136/practneurol-2017-001609 28626021

[B74] The Global Poliovirus Eradication Initiative [GPEI] (2018). *Global Polio Eradication Initiative.* Available at http://polioeradication.org/where-we-work/polio-endemic-countries/ [accessed June 03, 2018] 10.1136/practneurol-2017-001609 28626021

[B75] Tokarz R. Firth C. Madhi S. Howie S. Wu W. Sall A. (2012). Worldwide emergence of multiple clades of enterovirus 68. *J. Gen. Virol.* 93(Pt 9) 1952–1958. 10.1099/vir.0.043935-0 22694903 PMC3542132

[B76] Tse M. Lane C. Mott K. Onlamoon N. Hsiao H. Perng G. (2009). ICAM-5 modulates cytokine/chemokine production in the CNS during the course of herpes simplex virus type 1 infection. *J. Neuroimmunol.* 213 12–19. 10.1016/j.jneuroim.2009.06.007 19589604 PMC2750088

[B77] Tyler K. (2015). Rationale for the evaluation of fluoxetine in the treatment of enterovirus d68-associated acute flaccid myelitis. *JAMA Neurol.* 72 493–494. 10.1001/jamaneurol.2014.4625 25775436 PMC5086417

[B78] Varghese R. Iyer A. Hunter K. Cargill J. Cooke R. (2015). Sampling the upper respiratory tract for enteroviral infection is important in the investigation of an acute neurological illness in children. *Eur. J. Paediatr. Neurol.* 19 494–495. 10.1016/j.ejpn.2015.03.009 25868937

[B79] Walid M. Mahmoud A. (2009). Successful treatment with intravenous immunoglobulin of acute flaccid paralysis caused by West Nile Virus. *Perm. J.* 13 43–46. 10.7812/TPP/09-028 20740088 PMC2911811

[B80] Wang G. Zhuge J. Huang W. Nolan S. Gilrane V. Yin C. (2017). Enterovirus D68 subclade B3 strain circulating and causing an outbreak in the United States in 2016. *Sci. Rep.* 7:1242. 10.1038/s41598-017-01349-4 28455514 PMC5430842

[B81] Washington State Department of Health (2016). *Communicable Disease Epidemiology. Acute Flaccid Myelitis and Poliomyelitis Reporting and Investigation Guideline.* Chesterfield: DCHS.

[B82] Wei W. Guo H. Chang J. Yu Y. Liu G. Zhang N. (2016). ICAM-5 Telencephalin is a functional entry. *Cell Host Microbe* 20 631–641. 10.1016/j.chom.2016.09.013 27923705

[B83] Williams C. Thomas R. Pickersgill T. Lyons M. Lowe G. Stiff R. (2016). Cluster of atypical adult Guillain-Barré syndrome temporally associated with neurological illness due to EV-D68 in children, South Wales, United Kingdom, October 2015 to January 2016. *Euro Surveill.* 21:30119. 10.2807/1560-7917.ES.2016.21.4.30119 26848143

[B84] World Health Organization [WHO] (2018). *Poliomyelitis.* Available at: http://www.who.int/mediacentre/factsheets/fs114/en/ [accessed April 11, 2018]

[B85] World Health Organization [WHO] (2012). *(Emergencies Preparedness, Response) Severe Complications of Hand, Foot and Mouth Disease (HFMD) Caused by EV-71 in Cambodia – Conclusion of the Joint Investigation.* Available at: http://www.who.int/csr/don/2012_07_13/en/. [accessed April 11, 2018]

[B86] Xiang Z. Xie Z. Liu L. Ren L. Xiao Y. Paranhos-Baccalà G. (2016). Genetic divergence of enterovirus D68 in China and the United States. *Sci. Rep.* 6:27800. 10.1038/srep27800 27278628 PMC4899779

